# Antioxidant Status and Immune Activity of Glycyrrhizin in Allergic Rhinitis Mice

**DOI:** 10.3390/ijms12020905

**Published:** 2011-01-26

**Authors:** Xiao-Lan Li, Ai-Guo Zhou, Li Zhang, Wei-Jun Chen

**Affiliations:** 1 Ear-Nose-Throat (ENT) Department of the 1th Hospital Associated to the ShiHeZi University, ShiHeZi city, XinJiang 832008, China; 2 Acute Surgery Department of the 1th Hospital Associated to the ShiHeZi University, ShiHeZi city, XinJiang 832008, China; E-Mail: zhouag3@yahoo.com.cn; 3 The Department of Traditional Chinese Medicine of the 1th Hospital Associated to the ShiHeZi University, ShiHeZi city, XinJiang 832008, China; E-Mail: zhanglshu@yahoo.com.cn; 4 Medicament Section of the 1th Hospital Associated to the ShiHeZi University, ShiHeZi city, XinJiang 832008, China; E-Mail: chenwjshu@yahoo.com.cn

**Keywords:** allergic rhinitis, glycyrrhizin, antioxidant, immunity

## Abstract

Oxidative stress is considered as a major risk factor that contributes to increased lipid peroxidation and declined antioxidants in some degenerative diseases. Glycyrrhizin is widely used to cure allergic diseases due to its medicinal properties. In the present study, we evaluated the role of glycyrrhizin on lipid peroxidation and antioxidant status in the blood and nasal mucosa of allergic rhinitis (AR) mice. Mice were divided into six groups: normal control mice, model control (MC) mice, three glycyrrhizin-treated mice groups and lycopene-treated mice. Sensitization-associated increase in lipid peroxidation was observed in the blood and nasal mucosa of MC mice. Activities of antioxidant enzymes like superoxide dismutase (SOD), catalase (CAT), glutathione peroxidase (GSH-Px), total antioxidant capacity (TAOC) and levels of glutathione (GSH) were found to be significantly decreased in the blood and nasal mucosa in MC mice when compared to normal control mice. However, normalized lipid peroxidation and antioxidant defenses were reported in the glycyrrhizin-treated and lycopene-treated mice. Moreover, glycyrrhizin treatment still enhanced IFN-γ and reduced IL-4 levels in glycyrrhizin-treated mice. These findings demonstrated that glycyrrhizin treatment enhanced the antioxidant status and decreased the incidence of free radical-induced lipid peroxidation and improved immunity activities in the blood and nasal mucosa of AR mice.

## 1. Introduction

Allergic rhinitis (AR) affects both adolescents and younger children. AR may be regarded by parents as an irritation rather than as a significant disease and is often under-diagnosed and under-treated [1]. It is now recognized that AR is made up of more than the classic symptoms of sneezing, rhinorrhea, and nasal obstruction. AR is associated with impairments in how patients function in day-to-day life at home, at work, and in school [2,3]. Patients may also be bothered by sleep disorders, emotional problems, impairment in activities, and social functioning [4]. As allergic AR is one of the most frequent diseases encountered in clinical practice, the cost implications to society are enormous [5].

It has been suggested that oxidative stress is a crucial event in perennial AR [6–9]. Oxidative stress has been incriminated as one of several mechanisms that have induced toxic effects in different organs due to enhanced production of oxygen free radicals. Glycyrrhizin is a triterpene glycoside. It is a major active constituent of licorice root (*Glycyrrhiza glabra*) and has been attributed numerous pharmacological effects like anti-inflammatory, anti-viral, anti-tumor, antioxidant and hepatoprotective activities [10,11]. Recently, Glycyrrhizin has been reported with many immunomodulatory effects such as decreasing the recurrent attack rate of AR and asthma, improving semen quality with treatment [12], and modulating allergic inflammation in a murine model for asthma [13].

In Japan, glycyrrhizin injections have been used as a therapeutic drug for allergy inflammation since 1948 and for chronic hepatitis since 1979. Ram *et al.* [14] demonstrated that Glycyrrhizin significantly reduces OVA-induced airway constriction, airway hyperreactivity to methacholine and decreases lung inflammation including marked eosinophil infiltration in the mouse model of asthma. In addition, it reduces OVA specific IgE levels in serum and Th2 cytokines, IL-4 and IL-5 in BAL fluid. Glycyrrhizin has been proven to have an important potential in activating certain immune functions, such as induction of IFN production, augmentation of NK cell activity and modulation of the growth response of lymphocytes through augmentation of IL-2 production [15]. Dai *et al*. [16] investigated the effects of glycyrrhizin on the production of IL-12 and other cytokines, and found that it enhanced both IL-12 messenger RNA accumulation and protein secretion by peritoneal macrophages in response to lipopolysaccharide.

The objective of this study was to determine the antioxidant and immunomodulatory effects of Glycyrrhizin in AR mice, which would be beneficial to AR drug therapy.

## 2. Materials and methods

### 2.1. Preparation of Glycyrrhizin

The dried roots of licorice (600 g) were coarsely grounded and extracted with boiling water (4 L × 5) according to Nokhodchi’s mothod [17]. The combined solutions were filtered through a sterile 0.20 μm filter and were then boiled for 10 min and set aside at room temperature for 12 h. The solution was filtered again to eliminate albuminoid substances. After this time the solution was acidified gradually with sulfuric acid (20% v/v) step-by-step until no precipitation occurred. The yellow precipitate (∼27 g) was separated by centrifugation and was re-dissolved in a small amount of ammonium hydroxide, then its volume was adjusted to 290 mL with distilled water. The precipitation procedure with sulfuric acid was repeated. The precipitated material was rinsed several times with water in order to neutralize the sulfuric acid. The pasty, acid insoluble precipitate was dried at 40 °C and then ground as fine powder. The obtained sample was analyzed with HPLC and the purity of the resultant glycyrrhizin was 93%.

### 2.2. Animals

Male, Kunming mice (28–33 g, body weight, BW), 2–3 month-old, were kept in polyacrylic cages (38 cm × 23 cm × 10 cm) with ten animals per cage and housed in a room under controlled temperature (24–26 °C), relative humidity (50–60%); with 12 h light–dark cycles. Mice had *ad libitum* access to food in form of dry pellets and water.

### 2.3. Allergic Rhinitis Model

Mice were sensitized by subcutaneous injection (sc) with 1 mg antigen ovalbumin mixed with 80 mg aluminum hydroxide adjuvant in 1 mL saline at footpad, neck, back and groin on the first day. Local challenge was performed everyday from day 20 to day 26 by dripping ovalbumin in physiological saline (2 mg mL^−1^, 10 μL) into the bilateral nasal cavities using a micropipette. Mice in normal control were dripped saline. All procedures were in accordance with China’s National Animal Care Guidelines.

### 2.4. Animal Grouping and Treatment

Mice were randomly divided into six groups: the normal control, model, lycopene 20 mg kg^−1^ (as positive control drug) group, glycyrrhizin 10, 20, 30 mg kg^−1^ group. After the sensitization day 14, lycopene (30 mg/kg BW) and glycyrrhizin (40 and 80 mg/kg BW) were given orally for 20 days once a day. Mice in the normal control and model groups were given saline orally for 20 days once a day.

### 2.5. Blood and Tissue Samples

After 20 days of experiment and overnight fasting, six rats from each group were anesthetized with sodium pentobarbital (60 mg/kg body weight) and weighed. Blood was collected from the abdominal aorta into tubes containing EDTA, and plasma was prepared by low-speed centrifugation (1000 g for 20 min, 4 °C). Nasal mucosa were removed immediately, rinsed with cold saline, weighed and stored at −70 °C until use. Tissues were immediately homogenized in cold 10 mM Tris-HCl, pH 7.5 (1/10 w/v) with 10 up-and-down strokes at approximately 1200 rev/min in a Teflon-glass homogenizer. The homogenates were centrifuged at 4000 × g for 10 min to yield a clear supernatant fraction that was used for the biochemical analysis.

### 2.6. Measurement of OVA-Specific IgE in Plasma

For OVA-specific IgE titration, plates were coated with OVA (20 μg/mL) and biotin-conjugated rat mAb to mouse IgE (clone RME-1) were used as detection antibodies. Levels of OVA-specific antibodies were compared with IgE standards with predetermined concentrations (IgE = 1 μg/mL). The concentration of standard serum was arbitrarily assigned as 1 ELISA unit (1 EU).

### 2.7. Biochemical Analysis

The malonaldehyde (MDA) concentrations were determined using the method described by Jain *et al*. [18] based on TBA reactivity. The GSH concentrations were measured using the method described by Beutler *et al*. [19].

The catalase (CAT) activity was determined according to the Aebi method [20]. The rate of H_2_O_2_ decomposition was followed by monitoring absorption at 240 nm. One unit of CAT activity is defined as the amount of enzyme required to decompose 1 μmoL of hydrogen peroxide in 1 min. The enzyme activity was expressed as μmoL H_2_O_2_ consumed/min/mg protein. Superoxide dismutase (SOD) activity was estimated according to the method of Beauchamp and Fridovich [21]. The developed blue color in the reaction was measured at 560 nm. Units of SOD activity were expressed as the amount of enzyme required to inhibit the reduction of NBT by 50% and the activity was expressed as U/mg protein. Activity of glutathione peroxidase (GSH-Px) was determined according to the method of Lawrence and Burk [22]. The assay mixture consisted of 2.0 mL of 75 mM phosphate buffer (pH 7.0), 50 μL of 60 mM glutathione, 0.1 mL of 30 units/mL glutathione reductase, 0.1 mL of 15 mM EDTA, 0.1 mL of 3 mM NADPH and the appropriate amount of tissue supernatant to a final volume of 3.0 mL. The reaction was started by the addition of 0.1 mL of 7.5 mM H_2_O_2_. The rate of change of absorbance during the conversion of NADPH to NADP^+^ was recorded spectrophotometrically at 340 nm for 3 min. GSH-Px activity for tissues was expressed as μmoles of NADPH oxidized to NADP^+^ min^−1^ mg^−1^ protein. The antioxidant activities (TAOC) in serum and in tissue homogenates were determined with enzymatic methods using corresponding diagnostic kits (Nanjing Jiancheng Bioengineering Institute, Nanjing, China), according to the instructions of the manufacturer.

The IFN-γ level was measured by immunoassay. IL-4 level was measured using IL-4 ELISA Kit from Diaclone Research, France.

### 2.8. Statistical Analyses

Data obtained were expressed as mean ± SD (n = 10). The significance of differences between means of the TAA determined were examined by one-way ANOVA using the general linear model procedure of Statistical Analysis Systems statistical software package version 6.11 (SAS Institute, Cary, NC, USA). Differences with P < 0.05 were considered statistically significant.

## 3. Results

In untreated AR mice, sneezing and thin nasal discharge was accompanied by excessive scratching. In glycyrrhizin-treated groups (20 and 30 mg/kg BW), abnormal symptoms were not found. However, in AR mice treated with glycyrrhizin (10 mg/kg BW), sneezing and thin nasal discharge was accompanied by slightly scratching. In the lycopene-treated group, sneezing and thin nasal discharge was accompanied by slightly scratching. During the experiment, there was no serious or purulent nasal discharge at all. None of experimental animals developed secondary infections or were treated by additional drugs, such as antibiotics. In the medicine-treated groups, two mice died.

As can be seen in Table 1, body weights were significantly higher (*P* < 0.05) in the untreated AR mice than the normal control mice (*P* < 0.01). Mice having received an oral administration of lycopene and glycyrrhizin had lower body weight than that of the untreated AR mice. However, a statistical difference was not found. In addition, mice having received an oral administration of lycopene (20 mg kg^−1^ BW) had slightly (*P* > 0.01) lower body weight than that of mice receiving an equivalent dose of glycyrrhizin.

As can be seen from Table 2, OVA specific IgE levels were significantly higher (*P* < 0.05) in untreated AR mice than normal control mice (*P* < 0.01). Oral administration of glycyrrhizin significantly and in a dose-dependent manner (*P* < 0.05; *P* < 0.01) reduced OVA specific IgE levels in untreated AR mice compared to normal control mice. Oral administration of lycopene slightly (*P* > 0.05) reduced OVA specific IgE levels in untreated AR mice compared to normal control.

As can be seen from Table 3, while blood and nasal mucosa MDA contents were significantly higher in untreated AR mice than normal control mice (*P* < 0.01), they decreased significantly (*P* < 0.05, *P* < 0.01) in medicine-treated (lycopene and glycyrrhizin) groups when compared to untreated AR mice (*P* < 0.01). Mice having received an oral administration of lycopene (20 mg kg^−1^ BW) had markedly (*P* < 0.01) lower concentrations of MDA than that of mice receiving an equivalent dose of glycyrrhizin.

Table 4 shows the blood and nasal mucosa GSH levels at the end of the experiment. After 12 days of treatment, the GSH concentrations of untreated AR mice showed a significant (*P* < 0.01) decrease compared with the normal control mice. However, an increase of GSH concentration of mice in the medicine-treated (lycopene and glycyrrhizin) groups was observed (*P* < 0.01). There was a dose-dependent relationship between GSH concentration and glycyrrhizin consumption. Mice having received an oral administration of lycopene (20 mg kg^−1^ BW) had higher concentrations of GSH than that of mice receiving an equivalent dose of glycyrrhizin.

The SOD, CAT, GSH-Px and TAOC activities significantly (*P* < 0.01) decreased in blood and nasal mucosa of untreated AR mice compared to normal control mice. The decreases were significantly restored (*P* < 0.01) in the mice supplemented with lycopene and glycyrrhizin (Tables 5 and 6). There was a dose-dependent relationship between antioxidant enzymes’ activities and glycyrrhizin consumption. Mice having received an oral administration of lycopene (20 mg kg^−1^ BW) had higher antioxidant enzyme activities than that of mice receiving an equivalent dose of glycyrrhizin.

Table 7 shows the nasal mucosa IFN-γ, IL-4 levels and IFN-γ/IL-4 at the end of the experiment. After 12 days of treatment, the IFN-γ, IL-4 concentrations of untreated AR mice showed a significant (*P* < 0.01) decrease and increase compared with the normal control mice. Subsequently, IFN-γ/IL-4 markedly decreased. However, lycopene and glycyrrhizin administration significantly (*P* < 0.01) increased nasal mucosa IFN-γ and reduced IL-4 concentration of mice in the medicine-treated (lycopene and glycyrrhizin) groups. Subsequently, IFN-γ/IL-4 markedly increased. There was a dose-dependent relationship between IFN-γ, IL-4 levels and glycyrrhizin consumption. Mice having received an oral administration of lycopene (20 mg kg^−1^ BW) had lower IFN-γ and higher IL-4 levels than those of mice receiving an equivalent dose of glycyrrhizin.

## 4. Discussion

Oxidative stress plays an important role in allergic disorders and increased levels of oxidants are considered as markers of the inflammatory process. Overproduction of oxygen free radicals, while the natural scavenging mechanisms are weakened, is a process that is implicated in cell damage and multiorgan failure [23].

In the present study, we found a significantly lower plasma and nasal mucosa total antioxidant status in untreated AR mice. Our observation is supported by earlier reports [24]. Glycyrrhizin is used as natural sweetener, anti-ulcerative, and anti-inflammatory preparations [25]. Glycyrrhizin is reported to exhibit antiviral [26], anti-HIV [27,28], antitumor [29], antioxidant [30] and immunomodulatory activity [27,29]. Synthesis of glycyrrhizin or its analogues has not been proven to be commercially feasible [31]. Studies conducted earlier with glycyrrhizin have shown it to counteract CCl_4_-induced hepatotoxicity with the anti-oxidative potential [32]. As a major component of licorice root, glycyrrhizin consists of glycyrrhetic acid and two molecules of glucuronic acid. It has been shown that glycyrrhizin and its biologically transformed metabolite called 18 β-glycyrrhetinic acid, an aglycon component of glycyrrhizin, inhibit the passive cutaneous anaphylaxis and skin contact inflammation in mice models of contact hypersensitivity [33]. Lycopene is the red pigment in tomatoes and is known as a potent antioxidant [34,35]. Dietary intake of tomatoes and tomato products is reportedly associated with a decreased risk of cardiovascular disease and several types of cancer, especially prostate cancer [36–38].

The treatment of animals with glycyrrhizin significantly ameliorated GSH content with concomitant decrease in MDA. It can be suggested that glycyrrhizin has established antioxidant properties that might have counteracted the oxidative injury in AR mice by effectively scavenging and blocking the conjugation of reactive intermediates to GSH, as evident from ameliorated GSH content and decreased MDA formation in a dose dependent manner. Concomitant decreases were observed in GPx, CAT, TAOC and SOD activities in AR mice. Treatment with glycyrrhizin and Lycopene led to significant increases in the above mentioned enzymes. Our results indicated that the use of glycyrrhizin in the therapy of AR allows oxidative stress to be abolished or reduced by correcting the antioxidant system. It is evident from the results that glycyrrhizin induces a variety of enzymes; phase II enzymes involved in the detoxification and excretion of carcinogenic or toxic substances and other antioxidant enzymes responsible for maintaining a balanced state between free radicals/oxidants and the antioxidants within the cellular environment. In addition, blood IFN-γ level was markedly reduced, while IL-4 level increased in AR mice. Treatment with glycyrrhizin led to a significant increase of IFN-γ level and decrease of IL-4 level in medicine-treated mice. IFN-γ, or type II interferon, is a cytokine that is critical for innate and adaptive immunity against viral and intracellular bacterial infections and for tumor control. Aberrant IFN-γ expression is associated with a number of autoinflammatory and autoimmune diseases [39]. Interleukin-4, abbreviated IL-4, is a cytokine that induces differentiation of naive helper T cells (Th0 cells) to Th2 cells. Upon activation by IL-4, Th2 cells subsequently produce additional IL-4. It has many biological roles, including the stimulation of activated B-cell and T-cell proliferation, and the differentiation of CD4^+^ T-cells into Th2 cells [40]. Treatment with glycyrrhizin may induce Th1 cells differentiation, maturation and IFN-γ production. In addition, it reduced lgE synthesis possibly by inhibiting Th1 cells differentiation, maturation and IL-4 production, subsequently prevented AR development. Likewise, Lycopene increased IFN-γ level and decrease IL-4 level in medicine-treated mice. Lee *et al* [41] reported that Lycopene reduced the increased levels of IL-4 in OVA-challenged mice. This is in agreement with our results. Lycopene reduced the increased levels of GATA3 mRNA in OVA-challenged mice. This suggests that lycopene treatment is a novel, selective way to simultaneously suppress GATA-3 and increase T-bet expression in asthmatic reactions *in vivo* [42]. In the human body, glycyrrhizin is hydrolysed to glycyrrhetinic acid, which has a triterpenoid structure that is similar to the hormones of the adrenal cortex. Recently, research has shown that glycyrrhetinic acid inhibits the enzyme 11-beta-hydroxy steroid dehydrogenase, which is responsible for converting cortisol—the active form—into its inactive metabolites. Thus, inhibition of the enzyme by glycyrrhetinic acid significantly increases the levels of cortisol and also stimulation of the glucocorticoid receptors. This in turn potentiates the action of hydrocortisone, the main glucocorticoid secreted by the adrenal cortex. Hydrocortisone is associated with, and accounts for glyrrhizin and glycyrrhetinic acid’s antiinflammatory, anti-allergic and anti-arthritic effects, and also its role in stimulating the adrenal cortex after steroid therapy [43–45]. Fangzhibiyan tablets (produced by Guangxi Yunling Pharmaceutical Co. Ltd) can effectively alleviate patients suffering from AR. Glycyrrhizin is an important functional component in the tablet [46]. In short, treatment with glycyrrhizin may be considered as a therapeutic option in AR patients by, for example, increasing the antioxidant capacities, counteracting the oxidative injury and/or improving the control of the inadequate inflammatory and immune response.

## 5. Conclusion

As far as we know this is the first work that shows antioxidant and immunity effects of glycyrrhizin in AR mice. In conclusion, in AR mice glycyrrhizin consumption decreases blood and nasal mucosa antioxidant enzymes activities, lipid peroxidation and GSH levels, and enhances IFN-γ, reduces IL-4 levels, thus protecting the nasal mucosa oxidative injury and improving immunity activity in AR mice. The inhibition effects of the glycyrrhizin against nasal mucosa oxidative injury increase with increasing concentrations. Further studies are required to determine the causes behind the distinct effects of glycyrrhizin.

## Figures and Tables

**Table 1.** Body weight in different groups.

**Group**	**Body weight (g)**
NC	38.47 ± 1.17
MC	36.88 ± 2.13 a
lycopene 20 mg kg^−1^	37.32 ± 1.65
glycyrrhizin 10 mg kg^−1^	37.52 ± 1.93
glycyrrhizin 20 mg kg^−1^	37.83 ± 2.31
glycyrrhizin 30 mg kg^−1^	37.94 ± 2.51

a *P* < 0.01, compared with NC group.

NC: normal control; MC: model control.

**Table 2.** OVA specific IgE levels in different groups.

**Group**	**OVA specific IgE**
NC	1.35 ± 0.12
MC	4.59 ± 0.36 a
lycopene 20 mg kg^−1^	4.03 ± 0.44
glycyrrhizin 10 mg kg^−1^	3.49 ± 0.35 a
glycyrrhizin 20 mg kg^−1^	2.82 ± 0.17 b
glycyrrhizin 30 mg kg^−1^	1.98 ± 0.13 b

a *P* < 0.01, compared with NC group;

a *P* < 0.01,

b *P* < 0.01, compared with MC group.

**Table 3.** Blood and nasal mucosa MDA levels in different groups.

**Group**	**blood**	**nasal mucosa**

	**MDA (nmol/mg protein)**	**MDA (nmol/mg protein)**
NC	5.34 ± 0.09	4.93 ± 0.09
MC	7.85 ± 0.08 b	8.35 ± 0.13 b
lycopene 20 mg kg^−1^	5.12 ± 0.09 d	5.18 ± 0.03 d
glycyrrhizin 10 mg kg^−1^	7.15 ± 0.06 c	6.76 ± 0.14 d
glycyrrhizin 20 mg kg^−1^	6.98 ± 0.07 d	6.01 ± 0.12 d
glycyrrhizin 30 mg kg^−1^	6.77 ± 0.08 d	5.34 ± 0.12 d

b *P* < 0.01, compared with NC group;

c *P* < 0.01,

d *P* < 0.01, compared with MC group.

**Table 4.** Blood and nasal mucosa GSH levels in different groups.

**Group**	**blood**	**nasal mucosa**

	**GSH (nmol/mg protein)**	**GSH (nmol/mg protein)**
NC	8.04 ± 0.32	5.43 ± 0.11
MC	3.32 ± 0.11 b	2.97 ± 0.09 b
lycopene 20 mg kg^−1^	7.57 ± 0.12 d	6.03 ± 0.11 d
glycyrrhizin 10 mg kg^−1^	5.76 ± 0.15 d	3.89 ± 0.08 d
glycyrrhizin 20 mg kg^−1^	6.43 ± 0.18 d	4.38 ± 0.09 d
glycyrrhizin 30 mg kg^−1^	7.71 ± 0.24 d	5.96 ± 0.12 d

b *P* < 0.01, compared with NC group;

d *P* < 0.01, compared with MC group.

**Table 5.** Blood SOD, CAT, GSH-Px and TAOC activities in different groups.

**Group**	**SOD (U/mg protein)**	**CAT (U/mg protein)**	**GSH-Px (U/mg protein)**	**TAOC (U/mg protein)**
NC	198.4 ± 4.98	23.09 ± 0.98	15.02 ± 0.76	10.87 ± 0.35
MC	154.2 ± 7.34 b	17.56 ± 0.89 b	9.03 ± 0.09 b	7.45 ± 0.42 b
lycopene 20 mg kg^−1^	253.6 ± 9.04 d	23.98 ± 0.16 d	18.85 ± 0.13 d	12.09 ± 0.35 d
glycyrrhizin 10 mg kg^−1^	160.4 ± 5.09 d	19.85 ± 0.95 d	11.46 ± 0.72 d	8.98 ± 0.29 d
glycyrrhizin 20 mg kg^−1^	169.8 ± 4.99 d	21.12 ± 1.08 d	12.54 ± 0.58 d	9.34 ± 0.27 d
glycyrrhizin 30 mg kg^−1^	175.9 ± 5.07 d	22.11 ± 0.91 d	13.27 ± 0.29 d	10.32 ± 0.16 d

b *P* < 0.01, compared with NC group;

d *P* < 0.01, compared with MC group.

**Table 6.** Nasal mucosa SOD, CAT, GSH-Px and TAOC activities in different groups.

**Group**	**SOD (U/mg protein)**	**CAT (U/mg protein)**	**GSH-Px (U/mg protein)**	**TAOC (U/mg protein)**
NC	231.5 ± 7.7	24.65 ± 0.67	19.05 ± 0.57	11.05 ± 0.37
MC	165.3 ± 4.9 b	13.47 ± 0.54 b	11.87 ± 0.39 b	5.89 ± 0.04 b
lycopene 20 mg kg^−1^	227.4 ± 8.2 d	22.13 ± 1.09 d	21.03 ± 0.27 d	11.32 ± 0.09 d
glycyrrhizin 10 mg kg^−1^	179.4 ± 5.1 d	16.84 ± 0.78 d	14.07 ± 0.13 d	7.07 ± 0.08 d
glycyrrhizin 20 mg kg^−1^	195.6 ± 8.9 d	18.03 ± 0.67 d	16.48 ± 0.24 d	8.25 ± 0.07 d
glycyrrhizin 30 mg kg^−1^	218.8 ± 6.2 d	19.36 ± 0.47 d	18.04 ± 0.65 d	9.03 ± 0.08 d

b *P* < 0.01, compared with NC group;

d *P* < 0.01, compared with MC group.

**Table 7.** Nasal mucosa IFN-γ, IL-4 levels and IFN-γ/IL-4 in different groups.

**Group**	**IFN-γ (pg/mg)**	**IL-4 (pg/mg)**	**IFN-γ/IL-4**
NC	74.38 ± 2.82	56.29 ± 1.23	1.33 ± 0.09
MC	68.56 ± 1.73 b	61.35 ± 1.95 b	1.07 ± 0.05 b
lycopene 20 mg kg^−1^	71.36 ± 1.71 d	59.09 ± 1.06 d	1.19 ± 0.04 d
glycyrrhizin 10 mg kg^−1^	70.21 ± 1.47 d	59.28 ± 1.11 d	1.18 ± 0.07 d
glycyrrhizin 20 mg kg^−1^	72.82 ± 2.02 d	58.32 ± 1.79 d	1.25 ± 0.06 d
glycyrrhizin 30 mg kg^−1^	73.99 ± 2.36 d	56.93 ± 1.37 d	1.31 ± 0.05 d

b *P* < 0.01, compared with NC group;

d *P* < 0.01, compared with MC group.
